# Task-Irrelevant Features in Visual Working Memory Influence Covert Attention: Evidence from a Partial Report Task

**DOI:** 10.3390/vision3030042

**Published:** 2019-08-27

**Authors:** Rebecca M. Foerster, Werner X. Schneider

**Affiliations:** 1 Neuro-Cognitive Psychology & Centre for Interdisciplinary Research (ZiF), & Cognitive Interaction Technology Cluster of Excellence (CITEC), Bielefeld University, P.O. Box 100131, D-33501 Bielefeld, Germany; 2 Neuro-Cognitive Psychology & Cognitive Interaction Technology Cluster of Excellence (CITEC), Bielefeld University, D-33501 Bielefeld, Germany

**Keywords:** covert attention, template, attentional capture, visual working memory, involuntary top-down control

## Abstract

Selecting a target based on a representation in visual working memory (VWM) affords biasing covert attention towards objects with memory-matching features. Recently, we showed that even task-irrelevant features of a VWM template bias attention. Specifically, when participants had to saccade to a cued shape, distractors sharing the cue’s search-irrelevant color captured the eyes. While a saccade always aims at one target location, multiple locations can be attended covertly. Here, we investigated whether covert attention is captured similarly as the eyes. In our partial report task, each trial started with a shape-defined search cue, followed by a fixation cross. Next, two colored shapes, each including a letter, appeared left and right from fixation, followed by masks. The letter inside that shape matching the preceding cue had to be reported. In Experiment 1, either target, distractor, both, or no object matched the cue’s irrelevant color. Target-letter reports were most frequent in target-match trials and least frequent in distractor-match trials. Irrelevant cue and target color never matched in Experiment 2. Still, participants reported the distractor more often to the target’s disadvantage, when cue and distractor color matched. Thus, irrelevant features of a VWM template can influence covert attention in an involuntarily object-based manner when searching for trial-wise varying targets.

## 1. Introduction

Representations we are keeping briefly in memory can influence how we allocate our attention towards objects in the environment [1,2,3,4,5,6]. Experimentally, this has been proven by several studies showing that objects matching a current visual working memory (VWM) representation capture our attention more strongly than objects that are currently not maintained in VWM [7,8,9,10,11,12,13]. Specifically, in dual-task studies, participants had to keep one or several objects in VWM for a later recognition task. During the retention interval, they had to perform an unrelated visual search task. The currently maintained VWM objects could reappear as target or distractor in the search display. While search targets matching the VWM representation decreased search times, matching distractors prolonged search times. This was interpreted as obligatory attentional capture by WM contents. However, a prolongation by VWM-matching distractors was not always observed [14,15,16,17]. It has been argued that attentional capture by VWM-matching objects is only elicited, when the VWM representation is currently in an active rather than an accessory state in VWM [2,17,18,19,20]. Whether the VWM representation will be active or accessory is determined by several task constraints [1,2,15], such as whether participants relate the two tasks and try, for instance, to find the memory-matching item, or use it to refresh their memory for the later recognition task. A major constraint may also be whether the search target changes from trial to trial and, therefore, also affords VWM capacity, or whether it is constant and can thus be recoded into a long-term memory (LTM) template [18,21].

As theories and empirical evidence suggest that VWM maintains objects in the form of bound rather than segregated features [4,22,23,24,25,26,27,28], the question arises whether irrelevant features of a VWM object representation bias attention involuntarily. Two studies applying the above described dual-task design reported evidence in favor of, as well as against, obligatorily object-based biasing from VWM [17,29]. In Experiment 4 of Olivers and colleagues [17], either the shape or the color of an object had to be maintained for later recognition. In an interim search task, distractors matched the memory cue either in color, in shape, in both, or in no feature. When distractors matched the feature of the memory template that was relevant for later recognition, search times were prolonged compared to the no-match baseline. However, no increment was found with distractors matching a feature not needed for later recognition. From this result, it could be concluded that it is possible to retain only the relevant feature of a VWM object, while the relevant feature can efficiently be ignored and does not bias attention. Thus, the features of a single VWM object representation might be stored separately. However, not finding such a biasing effect for a feature does not mean that this effect does not exist. As also stated by the authors, an object that matches a relevant VWM feature will capture attention more strongly than an object that matches an irrelevant VWM feature. Therefore, the relevant feature will influence search behavior more often in a measurable degree compared to the irrelevant feature, even if both features of the VWM object set bias signals in an involuntarily object-based manner.

Gao and colleagues [29] delivered the first proof of existence that also a currently irrelevant feature of a VWM object representation can influence search task performance. Participants saw a colored shape, the color of which they had to remember for a later match-to-sample test. In an interim search task, distractors matched the memory cue either in color, in shape, in both, or in no feature. Not only the memory-relevant color, but also the memory-irrelevant shape match, caused slower search times compared to the no match baseline. Thus, an item matching an irrelevant feature of a VWM object representation had influenced behavior and thus must have captured attention.

A problem with an active-passive WM status interpretation of the dual-task results is that it can never be predicted in advance, whether a VWM representation will be active or accessory during the interim search task or how many and which representations will be active (see the ongoing debate on whether and when multiple items can be active, e.g., [2,30,31,32]). Because the VWM object is not relevant for the interim search task, it does not need to be kept in an active format during search. On the other hand, the reappearance of the VWM features during search will not be unnoticed by the participants so that they will speculate about how the two tasks might be related and whether it might be useful to attend to the matching items [15]. Attending the matching item is probably indeed useful by means of refreshing the memory content for the later recognition task, even if the VWM feature always reappears as a distractor [33,34,35]. Thus, recoding of the VWM object into an accessory state could have caused the dissociation of the relevant and irrelevant feature in Olivers and colleagues [17]. In Gao and colleagues [29], however, the irrelevant feature must have been in an active format in order to capture attention. Such post-hoc speculations of whether the VWM representation was active or accessory are, of course, unsatisfactory.

Recently, we developed an experimental design that tests whether relevant, as well as irrelevant, features of a single VWM object bias attention by ensuring that this VWM object is essential for the ongoing task, thus having to be kept in an active format [36]. Specifically, instead of a dual-task design, we asked participants to perform only a single search task with a trial-by-trial varying target that had to be maintained in VWM. Using a trial-by-trial target instead of a constant target ensures that the search task is based on VWM rather than on LTM [21]. First, a colored shape was presented as a search cue. Participants were instructed that the shape of the cue defines the target, while the color has to be ignored. After a variable fixation interval, two objects were presented left and right from the fixation cross. One object matched the cue’s shape and thus was the target, while another object was used as the distractor. Participants’ task was to saccade directly to the shape-defined target. Depending on the experiment, the target or the distractor, both, or none could match the search cue’s irrelevant color. As the shape of the search cue is needed to direct the saccadic eye movement towards the shape-matching target, the search cue has to be kept in an active format during the only ongoing task. If the search cue is maintained in VWM as an object with bound features [22,26,28], this implies that also the color is kept in an active format and should influence attention (see also [25] for the distinction of active versus passive VWM).

Indeed, we found that targets, as well as distractors that matched the irrelevant color of the VWM template, were more often looked at than different colored targets and distractors. Matching distractors captured the eyes even in an experiment in which targets never matched the cue in the irrelevant feature, although participants were informed that only the distractor matched the cue’s color in half of all trials. As a covert shift of attention precedes every saccadic eye movement [37], we concluded that the template-matching color captured covert attention. Thus, not only the relevant, but also the irrelevant, feature of a currently active VWM template object sets an attentional bias signal, arguing for involuntarily object-based attentional biasing from VWM.

While the eyes can only aim at one specific saccade target at a time, covert attention can be distributed in parallel to multiple locations [38]. Our previous study showed that in case of overt selection, the attentional bias by the search-irrelevant VWM feature is sometimes so strong as to overpower the bias by the search-relevant VWM feature, resulting in a saccade towards the distractor. However, saccadic selection can be viewed as resulting from competition between potential target objects via attentional weights [25,39,40]. Considering recognition, it seems possible to process multiple objects from a single glance because covert attention can be allocated in parallel to multiple visual objects [6,41,42], even if they have to be acted on sequentially [43]. Thus, the question remains to be answered, whether items matching irrelevant VWM features would capture covert attention if no saccadic reaction is required. In addition, it has been shown that motor measures, such as reaction times, do not always yield the same results as perceptual measures, such as percentage of correct recognition [44,45], maybe because there can be a dissociation between selection for action and selection-for-object-recognition [46,47] (but see [48]). Although we investigated percentage of direct target saccades instead of reaction time as main dependent variable in our previous study, saccadic reactions need an immediate motor command. Thus, it is an open question whether the effect can be observed with a task affording only perceptual processing and no immediate motor response. Here, we used a letter-report task to answer both questions in two single-task experiments.

## 2. Experiment 1

In Experiment 1, it was investigated whether items matching a task-irrelevant feature of a VWM object representation will capture covert attention. On the basis of our previous study [36], we used colored real-world objects as stimuli. However, instead of saccading to the target object, participants in our trial-by-trial cued partial report task had to recognize a briefly presented letter inside the shape-defined target object. Specifically, the shape-defined identity of a colored real-world object cue indicated in each trial, in which of two following objects the target letter will appear. The letter within the other object was by definition a distractor letter. Then, participants had to keep fixation on a central cross before two colored objects appeared left and right from fixation, each containing a letter and followed by a mask. Participants were instructed to report the letter appearing within the shape-defined target object. The target, the distractor, both, or no object could match the cue’s irrelevant color. This manipulation allows to investigate whether the task-irrelevant color influences attentional biasing and, therefore, letter recognition performance.

### 2.1. Materials and Methods

#### 2.1.1. Participants

Sixteen participants (5 male and 11 female, mean age 25 years) recruited at Bielefeld University, Germany, took part in the experiment after having provided written informed consent. Participants were naïve with respect to the study’s purpose, reported normal or corrected-to-normal visual acuity, and were compensated with 8€ per hour. The study was approved by the Committee for Ethics at the Department of Psychology (2015-024) at Bielefeld University in advance, and conducted in accordance with the Declaration of Helsinki.

#### 2.1.2. Apparatus and Stimuli

**Figure 1:**
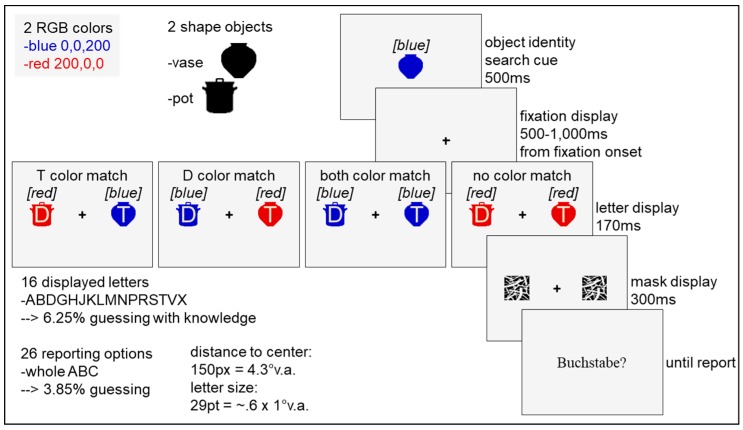
Material, procedure, and design of Experiment 1. Only the target (T color match), only the distractor (D color match), both (both color match), or no object (no color match) of the letter display matched the cue’s irrelevant color. The color words in brackets are added for greyscale printing. Neither the color words nor the condition names were present during the experiment. “Buchstabe” is the German word for letter. The schematic drawing is not true to scale.

Stimuli were displayed on a 19-in color monitor (View Sonic Graphics series G90fB, Brea, CA, U.S.) with a refresh rate of 100 Hz and a spatial resolution of 1024 × 768 pixels extending to 36 × 27 cm. Right gaze positions were recorded with an EyeLink 1000 desktop eye tracker (SR Research, Ottawa, ON, Canada) at 1000 Hz. A chin-and-forehead rest stabilized participants’ heads at a viewing distance of 71 cm. The experiment was programmed and run using SR Research’s Experiment Builder software on a Dell Precision T3600 with an NVIDIA GeForce GTX 970 graphics card. Luminance and color of all presented stimuli were measured at screen center in CIE Lxy coordinates with an X-Rite i1 Pro spectrophotometer. Stimuli were presented on a grey background (RGB 245, 245, 245; L = 97 cd/m^2^, x = 0.3, y = 0.3). A black plus (RGB 0, 0, 0; L = 0 cd/m^2^, x = 0.3, y = 0.3) with a size of 0.5 degrees of visual angle (dva) served as the central fixation marker. Real-world objects from the database of Konkle and colleagues ([49]; http://cvcl.mit.edu/MM/objectCategories.html) were modified using MATLAB R2013b so that they extended 1.4 dva. in foveal vision (49 pixels) and filled with a single color (Figure 1). In Experiment 1, either a vase or a pot could appear in blue (RGB 0, 0, 200; 14 cd/m^2^, x = 0.2, y = 0.1) or red (RGB 200, 0, 0; 25 cd/m^2^, x = 0.6, y = 0.3), and with one of 16 letters (A, B, D, G, H, J, K, L, M, N, P, R, S, T, V, X; Arial; font size 29 equaling to approximately 0.6 dva in width and 1.0 dva in height) superimposed on it in the background color (Figure 1). A set of four black and white pattern masks in the same size as the objects (1.4 dva) were used to terminate the letter display. The German word for “letter” signaled the start of the reporting interval. All letters of the whole alphabet could be typed in via a standard computer mouse keyboard. The cue indicating the target shape was always presented in the center of the screen. Target, distractor, and masks were located 4.3 dva (150 pixels) left and right from fixation.

#### 2.1.3. Procedure

The experiment started with a written instruction on the computer screen and a nine-point eye-tracking calibration and validation procedure. Then, a practice trial had to be passed that was not included in the analysis. Afterwards, the experimental trials started. Each trial began with a central colored real-world object presented for 500 ms indicating the shape-defined target object for the current trial. This cue was followed by a central plus that had to be fixated on (tolerance area of 2.5 dva around the center) for a randomly chosen duration between 500 and 1000 ms (uniform distribution). In case of successful fixation, the letter display came on containing one colored object left and one colored object right from fixation at a distance of 4.3 dva for 170 ms duration. A letter was superimposed on each object in the background color (Figure 1). One object was the same in terms of shape as the cue and thus the target object, while the other object was a distractor. Participants still had to keep central fixation while the letter display was on screen. If a participant did not manage to fixate on the central cross for the specified duration within a time out interval of five seconds or disengaged the fixation while the letter display was on screen, the trial was abandoned and repeated at a random position within the experimental block. In addition, calibration was repeated in this case. The letter display was terminated by a mask randomly chosen from the set of four masks presented at both the target and the distractor location for 300 ms duration. Finally, the German word for letter indicated to the participant to type in the target letter. There was no reaction time limit for the unspeeded letter report. Reporting a letter immediately started the next trial. The experiment consisted of 384 trials, separated in 4 blocks of 96 trials each. After each block, a feedback display informed participants about the number of completed and total blocks. Participants could start each block by pressing the space bar and were allowed to take breaks in-between.

#### 2.1.4. Design

The experiment consisted of four conditions (Figure 1). In the target color-match condition (T color match), the target object on which the target letter appeared matched the cue not only in its target-defining object shape, but also in its color. In the distractor color-match condition (D color match), the distractor object on which the distractor letter appeared was presented in the cue’s color. In the both color-match condition (T and D color match), the cue, the target, and the distractor object were of the same color. In the no color-match condition (no color match), the cue appeared in one of the two used colors, while both target and distractor object appeared in the other color. All combinations of conditions (4), locations (2), colors (2), and object identities (2) were equally often completed per block in random order. The same randomly chosen assignment of masks (one of four) and letter combinations (one of 16!) to the 384 trials was applied to all participants, but the randomly chosen trial order varied between participants.

#### 2.1.5. Analysis

Data were analyzed and plotted using Excel 2010 (Microsoft) and SPSS Statistics 22 (IBM). The dependent variables were the percentages of reported targets and distractors. Kolmogorov-Smirnoff tests did not reveal any deviation from normal distribution. Repeated measures analyses of variance (ANOVA) were used to analyze whether the dependent variables differed significantly between the four conditions. Investigating target letter reports reveals whether target object processing is influenced by the different color-match conditions. Investigating distractor letter reports reveals whether target-distractor confusion is influenced by the different color-match conditions. If sphericity was violated, Greenhouse-Geisser epsilon is reported along with uncorrected degrees of freedom. Planned paired *t*-tests were used to compare the dependent variables in the experimental conditions to the no-match baseline in case of a significant ANOVA. We decided to test the three match conditions to the no-match condition because we think that the lack of any color repetition serves the best baseline performance, i.e., performance based on the shape signal alone without any preference for selecting either object based on color. A chance level of 0.05 was applied to all analyses.

### 2.2. Results

Raw data is provided in the Supplementary Materials. On average 0.5 trials per participant had to be repeated because central fixation was not kept for the specified duration within five seconds. Further, 22.5 trials per participant had to be repeated because central fixation was disengaged while the letter display was on screen.

Table 1 provides descriptive values of target and distractor letter reports.

**Figure 2:**
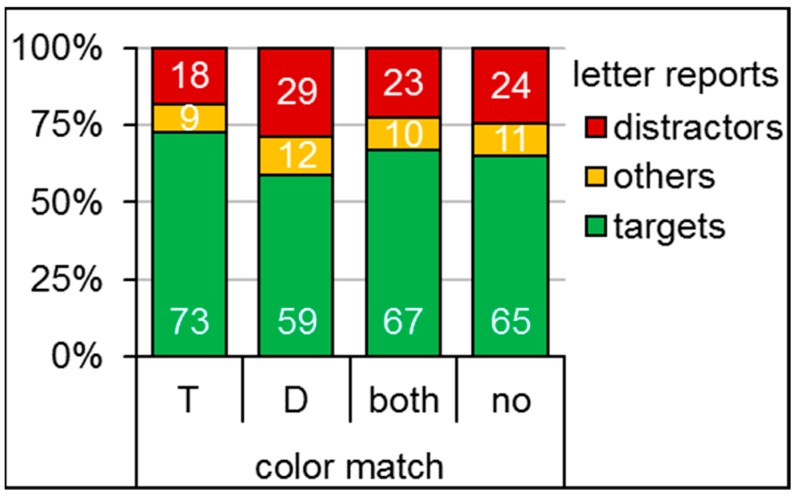
Mean percent of reported target (**green**), distractor (**red**), and other letters (**orange**) for the four color match conditions of Experiment 1: T = target color match, D = distractor color match, both = target and distractor color match, and no = no match of target and distractor to the cue’s color.

Repeated measures ANOVAs indicated significant differences across conditions for the percent of reported target letters (*F*(3, 45) = 9.19, *p* < 0.01, *η_p_*^2^ = 0.38, *ε* = 0.50), as well as reported distractor letters (*F*(3, 45) = 6.61, *p* < 0.01, *η_p_*^2^ = 0.31, *ε* = 0.54). Planned paired *t*-tests indicated that the effects were due to significantly more target letter reports (*t*(15) = 2.99, *p* < 0.01, *Cohen’s d_z_* = 0.75) and less distractor letter reports (*t*(15) = 2.63, *p* < 0.05, *Cohen’s d_z_* = 0.66) in case of the target color-match compared to the no-match baseline. Complementary, significantly less target letters (*t*(15) = 2.65, *p* < 0.05, *Cohen’s d_z_* = 0.66) and more distractor letters (*t*(15) = 2.14, *p* < 0.05, *Cohen’s d_z_* = 0.53) were reported in case of the distractor-match compared to the no-match baseline. Reports of the distractor letter (*t*(15) = 1.08, *p* = 0.30, *Cohen’s d_z_* = 0.27) did not differ significantly between the both-match and the no-match condition, while target letter reports were marginally more frequent in the both-match than in the no-match condition (*t*(15) = 1.99, *p* = 0.06, *Cohen’s d_z_* = 0.50). The mean values can be seen in Figure 2.

### 2.3. Discussion

In Experiment 1, participants reported the target letter most often and the distractor letter least often, when only the target object matched the search cue’s irrelevant feature. Complementary, participants reported the target letter least often and the distractor letter most often, when only the distractor object matched the search cue’s irrelevant feature. Thus, the irrelevant feature of the VWM representation of the search cue biased covert attention likewise towards target and distractor objects, arguing that also irrelevant features of the cue were enhanced via top-down control. However, target and distractor color matches were intermixed in Experiment 1, and participants did not know the exact probabilities. When noticing that the target sometimes appears in the cue color, participants might have nevertheless chosen to tune their attention to the cue’s color. Thus, it is possible that participants only choose to ignore the task-irrelevant cue color, if the target is never colored likewise. Even more so, if the cue color can only be present as a distractor, participants might even be able to strategically down-weigh the cue’s color, using it as a template for rejection [50,51]. Experiment 2 was conducted to investigate these two possibilities.

## 3. Experiment 2

Experiment 1 showed that covert attention was captured by a task-irrelevant feature of a VWM template object. Experiment 2 was conducted in order to reveal whether a distractor-defining feature of a VWM template object can be effectively ignored or even used as a template for rejection [50,51]. Again, participants had to report the letter appearing on that object that was identical in terms of shape to a previously presented colored cue. In contrast to Experiment 1, the target object on which the to-be-reported letter appeared never matched the cue’s color. Instead, the distractor object on which the to-be-ignored letter appeared matched the cue’s color in half of the trials. Additionally, participants were informed about this misleading nature of color.

### 3.1. Materials and Methods

#### 3.1.1. Participants, Materials, and Procedure

**Figure 3:**
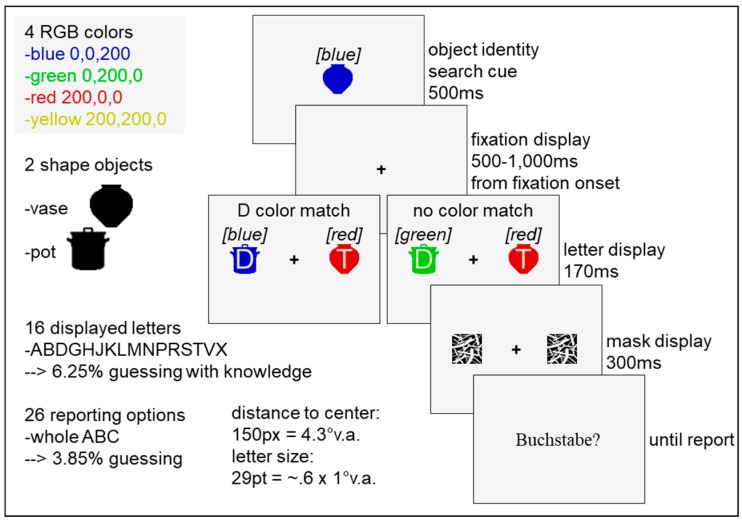
Material, procedure, and design of Experiment 2. Either only the distractor (D color match) or no object (no color match) of the letter display matched the cue in its irrelevant color. The color words in brackets are added for greyscale printing. Neither the color words, nor the condition names were present during the experiment. “Buchstabe” is the German word for letter. The schematic drawing is not true to scale.

A new sample of sixteen participants (7 male and 9 female, mean age 26 years) recruited at Bielefeld University, Germany, took part in Experiment 2 after having provided written informed consent. Again, participants were naïve with respect to the study’s purpose, reported normal or corrected-to-normal visual acuity, and were compensated with 8€ per hour. The same materials were used as in Experiment 1, except that the vase and the pot could appear in one of four colors (Figure 3), namely blue (RGB 0, 0, 200; 14 cd/m^2^, x = 0.2, y = 0.1), green (RGB 0, 200, 0; 74 cd/m^2^, x = 0.3, y = 0.6), red (RGB 200, 0, 0; 25 cd/m^2^, x = 0.6, y = 0.3), or yellow (RGB 200, 200, 0; 83 cd/m^2^, x = 0.4, y = 0.5). The procedure was exactly the same as in Experiment 1.

#### 3.1.2. Design and Analysis

**Figure 4:**
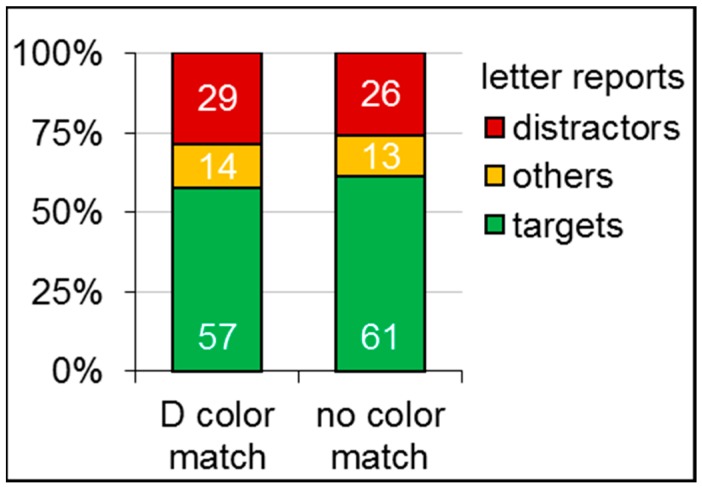
The plot shows mean percent of reported target (**green**), distractor (**red**), and other letters (**orange**) for the two color match conditions of Experiment 2: distractor (**D**) and no match with the cue’s irrelevant color.

Experiment 2 consisted of two conditions (Figure 4). In the distractor color-match condition (D color match), the distractor object on which the distractor letter appeared matched the cue’s color, while the cue’s color did not reappear in the no color-match condition (no color match). This time, target and distractor shape were always presented in distinct colors. Each combination of conditions (2), locations (2), and object identities (2) was completed equally often per block in random order. The same randomly chosen assignment of masks (one of four), letter combinations (one of 16!), and color combinations (one of four!) to the 384 trials was applied to all participants, whereby color combinations were balanced per experimental half, and trial order varied between participants. Again, data were analyzed using Microsoft Office’s Excel 2010 and IBM’s SPSS Statistics 22. The dependent variables were again the percentages of reported targets, distractors, and any other letters. None of them deviated significantly from the normal distribution due to Kolmogorov-Smirnoff tests. Paired *t*-tests compared the dependent variables between the distractor match and the no match condition. A chance level of 0.05 was applied.

### 3.2. Results

Raw data is provided in the Supplementary Materials. On average, 0.3 trials per participant had to be repeated because central fixation was not kept for the specified duration within five seconds. Further, 39.8 trials per participants (of the 384 trials) had to be repeated because central fixation was disengaged during the letter display. Planned paired *t*-test revealed that participants reported significantly less targets (*t*(15) = 4.88, *p* < 0.01, *Cohen’s d_z_* = 1.22) and more distractors (*t*(15) = 3.01, *p* < 0.05, *Cohen’s d_z_* = 0.75) in case of the distractor color match compared to the no-match baseline. Descriptive values are provided in Table 2 and Figure 4.

### 3.3. Discussion

In Experiment 2, participants reported more often the distractor instead of the target letter, when the distractor object matched the task-irrelevant color of the cue compared to the situation in which the cue’s color did not appear in the letter display. This was the case, although the target object never matched the cue’s color so that it is always dysfunctional to tune attention towards color-matching objects. The results of Experiment 2 thus indicate that the influence of the irrelevant feature of a trial-wise varying—and thus VWM-based—search template was not only object-based but also involuntary in nature that cannot be overcome by voluntary down-regulation.

## 4. General Discussion

In the present study, we investigated whether a target object whose representation should be kept in VWM for the consecutive search task (partial report) will bias covert attention in an involuntarily object-based manner. In order to test this hypothesis, we investigated the influence of a task-irrelevant feature of a trial-wise varying search-target cue within a trailing partial report task that required to report a letter inside a target object in an unspeeded manner. The target letter in the target object was always accompanied by one distractor letter in a distractor object. Specifically, a colored shape cue (search target) indicated to participants on which of the following shapes a to-be-reported letter will appear. Although participants were told that the color was uninformative (Experiment 1) or even distractor-defining (Experiment 2), they could not prevent attending towards color-matching items in the search display so that they occasionally reported the letter on the cue-colored distractor to the disadvantage of the target letter. Thus, participants could neither ignore color nor use it as a template for rejection [50,51]. Together, these results imply that covert attention is obligatorily biased towards objects matching an irrelevant feature of a trial-wise varying search (partial report) target arguing for involuntarily object-based top-down control of its VWM representation (cf. [9] for the terminology of “involuntary top-down”).

The results of the present study extend our previous finding that objects matching task-irrelevant features of a VWM object representation can capture the eyes—a proxy for covert attention [37]—in a visual search task [36]. While participants had to saccade to a target shape indicated by a colored shape template in our previous experiment [36], no overt shift of orienting was required here. Thus, the object-based bias from a VWM representation of a search target cannot only influence behavior when a single item had to be selected, such as a saccade target, but also when multiple items are competitively processed in parallel for access to VWM [5]. We argue within the TVA framework [6] that the increased attentional weight of a distractor matching an irrelevant feature of a VWM object reduces the relative attentional weight of the target, leading to a decrease in its report probability. In general, the results are perfectly in line with all theoretical approaches that assume competition for attention allocation based on a mixture of bottom-up and top-down feature weighting of all environmental objects, such as the biased competition theories [5,6,42], the Guided Search theory [52], or priority map models [53,54].

The results of the present study support the idea that VWM objects bias attention in an object-based manner. Results from dual-task studies investigating this question were inconclusive [17,29]. While participants were selectively biased towards search distractors matching relevant VWM features, but not irrelevant VWM features, of the same object in Experiment 4 of Olivers and colleagues [17], relevant and irrelevant VWM features of the same object captured attention likewise in Gao and colleagues [29]. Because both studies employed a dual-task design in which a visual search task was performed in the retention interval of a VWM recognition task, it can only be concluded in a post-hoc manner, whether the VWM representation was kept in an active format (biasing effect) or in a passive format (no biasing effect). As the VWM task was unrelated to the visual search task and the search target was constant, participants might have recoded the memory representation into an accessory state [2,17,18]. However, as the features of the VWM object could reappear during the search task, it is also possible that they might have kept the representation in an active format or even activated it by the matching items as a rehearsal process [34,35]. Differential VWM maintenance states might have caused the contradicting dual-task results. Alternatively, the different stimulus material could have been responsible for the conflicting results. It is known that the target-distractor similarity influences how well a target can be found among distractors [42,55]. Similarly, the target-distractor similarity seems to influence whether a distractor matching a VWM feature will have enough power to overrule the bias towards the target [56,57].

As stated above, the state of the VWM representation can often not be predicted in dual-task designs and is therefore inferred post-hoc from the results (active, if effect observed; accessory, if no effect observed). By our single-task design [36], we ensure that the relevant feature of the VWM template has to be kept always in an active state in order to find the target. If all features of the VWM object are maintained as bound features rather than segregated features [22,26], then the irrelevant feature should also be in an active state. Moreover, if all features of a currently active VWM object representation bias attention, then an object matching the irrelevant feature should capture attention. Indeed, in our partial report task, the task-irrelevant color of the VWM template captured attention as indicated by the decrement in target-letter report performance. Revealingly, the target was not only reported less often, when the distractor matched the template’s irrelevant color, but also the distractor of the same trial was reported more often instead. This result implies that the VWM representation influenced covert attention allocation in an object-based manner so that target-letter processing suffered due to distractor-letter processing, when the latter shared an irrelevant feature of the VWM template object. Even more so, participants more often confused which letter was actually the target and which the distractor so that they reported the distractor letter more often in this case, instead of guessing. This pattern of results was even found when the shape-defined target never matched the template’s color known by the participants (Experiment 2), arguing that object-based biasing from VWM is involuntary. The bias is object-based because the fact that both features belong to a single object in VWM seems to be crucial [18]. As one feature of the VWM template object defines the target in the ongoing task, the whole object with both features needs to be kept in an active VWM state. Holding the object in an active state in VWM is voluntary and thus top-down controlled, as is setting an attentional bias towards template-matching objects in the environment. This top-down control is actually required in order to preferentially attend towards environmental objects that match the task-relevant feature of the VWM object. The automatic co-activation of the irrelevant or even distractor-defining feature is, however, involuntary. Participants, thus, have the choice not to set any bias, which would lead to chance performance, or to set a bias towards both features of the template object, which allows to perform the task above chance in both conditions and causes capture by objects matching the irrelevant VWM feature. Note, however, that we do not make any claims about whether guidance by feature conjunctions is beneficial above what would be expected from the sum of their higher-weighted features. This is also an interesting question and has been investigated elsewhere [58,59,60]. It seems that guidance differs if the target is defined by a conjunction of features rather than identifiable on the basis of a single feature [60].

Although we think that an irrelevant feature of an active VWM object obligatorily elicits a bias signal, we also think that this does not guarantee a manifestation in observable behavior [56,57]. As is represented in our data, in the majority of trials, the relevant feature elicits the stronger bias signal resulting in adequate behavior (correct report). If the bias of the relevant feature had been even stronger, e.g., because the discriminability of target and distractor had been better in the relevant compared to the irrelevant feature [7,56,57], then an observable effect could have been diminished. This argumentation is in line with a recent review on the relation of attention and VWM [1].

Finally, we want to address four open questions on the issue of involuntary top-down control by search irrelevant features of a trial-by-trial varying search/partial report target. Firstly, while several EEG and fMRI studies showed that the attentional capture is reflected in neuronal activity in case of behavioral effects [61,62,63], no study so far has investigated whether the VWM-matching distractor also elicits neuronal activity in the absence of a behavioral manifestation. Thus, future studies have to test, whether brain activity (e.g., an N2pc) is elicited by the irrelevant feature of a currently relevant VWM object in a single-task design, even if a behavioral effect is absent, which would be predicted by our hypothesis of obligatorily object-based top-down control of all VWM features. Secondly, what might be the role of recoded verbal WM stimulus representation in modulating our type of attentional capture? We used a limited set of distinct colors and objects, which can easily be verbalized. Capture by more, as well as less, verbalizable VWM content of different set sizes and when searching for more and less verbalizable targets has been found in dual-task studies, with stronger capture by less verbalizable WM content [8,9,10,17,50]. Thus even stronger capture in experiments with less verbalizable features might also be observed in our paradigm. Thirdly, there was always only one distractor in our search display so that relative attentional weights were calculated only for one target and one distractor. Following the neuroscience-based biased competition framework of attention in general [5] and TVA [6] as a specific version of it, we think that adding just one distractor to a target creates competition that reduces the performance for the target report [64]. How the addition of more distractors influences target performance should also be dependent on factors such as target-distractor similarity or heterogeneity of distractors in the display [42]. Thus, it is an open question of how the addition of different distractors with different similarity to the target and to each other in terms of the relevant and the irrelevant feature would modulate the capture strength of the VWM-matching distractor. Moreover, distractor suppression from WM might also play a role in our studies, which has been shown to scale with distractor load and distractor heterogeneity [65]. Fourthly, adding load to VWM could reveal, on the one hand, how capture by the task-irrelevant feature is modulated by VWM load per se. On the other hand, this would allow the investigation of how features of additional VWM objects interact with the task at hand and compare that to the dual-task situation [31,66].

## Figures and Tables

**Table 1:** Means and standard deviations (in brackets) in percent of reported target and distractor letters for the four color-match conditions of Experiment 1.

Color-Match Condition	Target Letter Report	Distractor Letter Report
target	73 (17)	18 (14)
distractor	59 (19)	29 (17)
both	67 (18)	23 (15)
no	65 (17)	24 (14)

**Table 2:** Means and standard deviations (in brackets) in percent of reported target and distractor letters for the two color-match conditions of Experiment 2.

Color-Match Condition	Target Letter Report	Distractor Letter Report
distractor	57 (18)	29 (15)
no	61 (18)	26 (14)

## Supplementary Materials

The following are available online at https://www.mdpi.com/2411-5150/3/3/42/s1.
